# Adolescents’ use of online food delivery applications and perceptions of healthy food options and food safety: a cross-sectional study in the United Arab Emirates

**DOI:** 10.3389/fnut.2024.1385554

**Published:** 2024-04-02

**Authors:** Sheima T. Saleh, Tareq M. Osaili, Ayoub Al-Jawaldeh, Haydar A. Hasan, Mona Hashim, Maysm N. Mohamad, Salma Abu Qiyas, Haleama Al Sabbah, Rameez Al Daour, Radhiya Al Rajaby, Emad Masuadi, Lily Stojanovska, Dimitrios Papandreou, Antonis Zampelas, Ayesha S. Al Dhaheri, Hanin Kassem, Leila Cheikh Ismail

**Affiliations:** ^1^Department of Clinical Nutrition and Dietetics, College of Health Sciences, University of Sharjah, Sharjah, United Arab Emirates; ^2^Department of Nutrition and Food Technology, Faculty of Agriculture, Jordan University of Science and Technology, Irbid, Jordan; ^3^Regional Office for the Eastern Mediterranean (EMRO), World Health Organization (WHO), Cairo, Egypt; ^4^Department of Nutrition and Health, College of Medicine and Health Sciences, United Arab Emirates University, Al Ain, United Arab Emirates; ^5^Public Health Department, College of Health Sciences, Abu Dhabi University, Abu Dhabi, United Arab Emirates; ^6^Department of Public Health Institute, College of Medicine and Health Sciences, United Arab Emirates University, Al Ain, United Arab Emirates; ^7^Institute for Health and Sport, Victoria University, Melbourne, VIC, Australia; ^8^Department of Food Science and Human Nutrition, Agricultural University of Athens, Athens, Greece; ^9^Nuffield Department of Women’s & Reproductive Health, University of Oxford, Oxford, United Kingdom

**Keywords:** food applications, digital food environment, food choices, consumer perception of healthy food, adolescence

## Abstract

**Introduction:**

This cross-sectional study aimed to assess Online food delivery applications (OFDA) usage trends among adolescent users in the United Arab Emirates (UAE), focusing on their perceptions of healthy food options and food safety (*n* = 532).

**Methods:**

Sociodemographic information, frequency of OFDA use, factors affecting food choices, and perceptions of healthy food and food safety were investigated. A total perception score was calculated for each participant;

**Results:**

Most participants used OFDAs weekly (65.4%), favoring fast food (85.7%). Factors like appearance and price drove food choices (65.0%), while taste and cost hindered healthy food orders (29.7 and 28.2%). Younger and frequent users had lower scores for perceiving healthy food, while seeking healthy options was associated with higher scores (*p* < 0.05). Females and those seeking healthy food showed higher food safety scores (*p* < 0.05).

**Discussion:**

The study suggests tailored interventions to promote healthier choices and improve food safety perceptions among adolescents using OFDAs in the UAE.

## 1 Introduction

Adolescence entails a critical period of physical, mental, and social growth as a child transitions toward adulthood (1). During this period, adolescents learn and adopt habits and choices that tend to persist into adulthood (2, 3). According to global statistics, the prevalence of overweight and obesity among children and adolescents who are 5–19 years old has more than quadrupled from 4% in 1975 to over 18% in 2016 (4). Most recent data in the UAE indicates a 29 and 35% prevalence of overweight and obesity among 4-13-year-old and 13-19-year-old children and adolescents, respectively (5, 6). Higher estimates of overweight and obesity have been reported in studies among 13-17-year-old adolescents in the national Global School-based Student Health Survey, reaching up to 55% (7). These numbers represent a serious public health issue that needs to be tackled to curb future health consequences that can persist into adulthood (8).

Numerous external factors leading to obesity have changed over the past few decades, with higher availability and accessibility of more processed and energy-dense food. The global economic landscape has significantly increased purchasing power and food availability at the individual level. The rapid proliferation of supermarkets and the exponential growth of the fast-food sector are central to this transformation, posing a dramatic impact on global eating habits (9, 10). This change has shifted diets toward a considerable reliance on ultra-processed foods high in sugars and saturated fats, effectively becoming the predominant energy source in many countries (9, 10). The flood of extensively advertised, convenient, and relatively inexpensive ultra-processed meals has significantly increased the energy level of the food supply far beyond actual population requirements (10). This surplus and a lack of nutritional alternatives encourages a diet heavy in calories, saturated fats, and added sweets, predisposing people to weight gain and the start of obesity (11). Furthermore, advancements in technology and its increasing use among the youth, and the increasingly sedentary nature of daily life have also contributed to these increasing rates (12).

Several studies in the UAE provide evidence of unhealthy food choices among this age group. One study showed that 30% of 4-13-year-olds exceeded their estimated energy requirements (5). Other studies showed that adolescents now consume more food away from home, consume fewer fruits and vegetables, and do not engage in physical activity (6, 13, 14).

Making food choices as an adolescent results from the interaction between external environmental factors and personal factors (15). This period is distinguished by increasing autonomy and a shift from spending more time with their parents to spending it with friends and peers away from home (16), resulting in a more significant influence of peers on their food choices (17). Nonetheless, profound evidence indicates that adolescents’ exposure to the marketing of food and beverages that are high in energy, fat, and sugar and their engagement with unhealthy food products on social media could take a significant toll on increasing obesity rates (18, 19).

Food environments directly link to dietary habits and health consequences, including noncommunicable diseases and obesity (20, 21). In the modern world, the digitalization of food environments has led to novel forms of acquiring foods and beverages. These include online grocery shopping and food delivery through websites or smartphone applications (22). The extensive growth of information communication technology and smartphones and the development of mobile applications have indisputably penetrated people’s lifestyles (23). Among the most popular mobile applications downloaded are mobile OFDA and statistics show that the number of users for OFDA has more than doubled from 2017 to 2022, reaching 1850 million users worldwide (24).

Smartphone applications can provide a robust medium for adolescents to acquire and adopt healthy or unhealthy food choices. Using such applications allows consumers to access and place orders from a wide range of food outlets at their convenience and to track their ordered meal till it reaches their chosen destination (25). Recent data shows that certain features of OFDA support consumers’ intention to use them, including online reviews, restaurant ratings, tracking, and price value (26). Moreover, other factors extend to certain aspects of food safety and hygiene, referred to as food delivery hygiene, which can be defined as the ability of the delivery person or company to maintain the safety and cleanliness of food delivery services (27).

Although available data on OFDA use in the Middle East is scarce, limited data indicate that adult users exhibit unhealthy dietary practices (28) and that consumers perceive the online food environment as unhealthy (29).

With the influx of food delivery applications and their mere ease of use, especially among adolescents in the digital age, delving into their usage and perceptions could yield a deeper understanding of the potential factors that shape their food choices. This may further contribute to filling knowledge gaps on personal factors and perceptions of healthy food availability in the online food environment when accessibility to such platforms is especially easy for this group.

To the best of our knowledge, there is no available data on adolescents’ OFDA usage and perceptions in the UAE, and as such, this study aims to assess the trends of OFDA usage among adolescents and investigate their perception of healthy food options and food safety through these apps.

## 2 Methods

### 2.1 Study design and participants

A cross-sectional, web-based study was carried out between January and June 2023, targeting adolescent OFDA users residing in the UAE. The inclusion criteria were adolescents aged 10 to 19 years as per the World Health Organization definition (1) and those who use OFDA at least once per month. The study participants were recruited using a convenience sampling method, allowing for better accessibility to people who satisfied the inclusion criteria within a restricted timeframe.

A sample size of 461 adolescents was determined based on the following formula with a confidence interval of 95%:


$$N=z^{2}\times P\times \left(1-P\right)/e^{2}$$

Where z = 1.96; P = (estimated proportion of the population that presents the characteristic) = 0.5; e (margin of error) = 0.05; N (sample size) = 384 participants. An additional 20% was added to the required sample size to account for any non-response bias or incomplete data, resulting in a target sample size of 461 participants.

Given the convenience sampling methodology employed in the study, 10 schools were contacted via email to request permission for data collection, ensuring the inclusion of at least one school in each of the seven Emirates. Of these, only three schools agreed to participate in the study (one in each of the three highly populated emirates: Abu Dhabi, Dubai, and Sharjah). A web link connecting to the online survey was sent to the administration and distributed to the students. While efforts were made to approach a wide array of schools, the participation of schools was voluntary, leading to variations in the distribution across emirates based on their willingness to participate and student and parent engagement. In addition, the web link was also shared via email invitations, with the contact lists being gathered from personal and professional contacts of the research team, including colleagues, friends, and family members, to disseminate among the target group to ensure a wider distribution and recruitment of participants from other emirates.

Parental consent was obtained through an online informed consent form, where parents were provided with detailed information about the study and asked if they agreed to their child’s participation. Upon their approval, adolescents were provided with a simplified explanation of the study and asked to provide their consent assent before completing the survey. Participants were also informed that only one response would be accepted using the link. This restriction was enforced by the survey platform as only one response was allowed from each device to ensure data integrity. No personal information was collected during the survey to ensure confidentiality, and participants were assured that their responses would remain anonymous and would not affect their academic standing or relationships within the school community.

This study was conducted following the guidelines in the Declaration of Helsinki. All procedures involving human subjects/patients were approved by the University of Sharjah Research Ethics Committee (REC-22-02-16-09-S). The online form eased the consent process by presenting participants with an electronic consent form detailing the study’s goal, methods, potential risks, and benefits. Before advancing to the survey questions, participants were requested to indicate their affirmative approval by clicking a marked ‘I agree’ button. All participants provided an electronic written informed consent before answering the survey questions.

### 2.2 Survey questionnaire and data collection

The survey used in this study was adapted from a previous tool developed by the research team and validated for use among adults (30). The original survey included 27 close-ended questions using Likert-scale, dichotomous, multiple choice, and checklist format (30). In the present study, some questions were omitted, while others were included to tailor the questionnaire for the present study population. These adjustments aligned the survey with the experiences and demographic characteristics pertinent to adolescents. For instance, some of the questions added were if the mother works outside the home and if, when using OFDA, a healthy or homemade meal is usually available at home. These questions were added to gain insights into the participants’ experiences and the possible impact of having a mother working outside on the frequency of using the apps.

The final version of the survey used in this study consisted of five sections. The first section focused on sociodemographic information, asking about sex, age, the emirate of residence, daily allowance, and maternal employment status. The selection of these factors was essential to describe the study population and their influence on food habits and choices.

The second section inquired about OFDA usage trends and explored participants’ behavior, inquiring about the frequency of OFDA use, most frequently used apps, the predominant type of food ordered (culinary styles or preferences), factors affecting food choices, and whether participants looked for healthy food options. The following section explored participants’ perceptions of healthy food and their concerns when choosing healthy options. These questions were chosen for their direct relevance to understanding participants’ decision-making when ordering meals online.

The last two sections comprised seven questions in each section and were employed to understand participants’ perspectives on healthy food ordering, food safety, and delivery cleanliness using OFDA platforms. The questions used a 5-point Likert scale (response options: 1: strongly disagree, 2: disagree, 3: neutral, 4: agree, 5: strongly agree). Participant responses were recorded and were grouped into three categories (agree, neutral, and disagree) for descriptive analysis. In addition, a score ranging from 7 to 35 was calculated for each participant for inferential analysis.

### 2.3 Data analysis

Descriptive statistics, such as frequencies and percentages, were used to summarize the demographic characteristics of the participants, their OFDA use, perception of a healthy meal, and perceptions of healthy food ordering and food safety and hygiene via OFDA. Data distribution was assessed using the Shapiro–Wilk’s test, which indicated a non-normal distribution (*p* < 0.05). Therefore, non-parametric tests and median and interquartile ranges (IQR) were used to analyze and describe the data. The frequency of using OFDAs was categorized into a dichotomous variable where frequent use corresponds to response options: daily, 4–6 times/week, and 2–3 times/week; infrequent use corresponds to response options: once/week and once/month. A total healthy food perception score was calculated for each participant based on their responses to the seven perception items by summing their responses. The score could range from 7 to 35, with a higher score indicating a more positive perception toward healthy food on OFDA. A reverse scale was used for negative-worded items “I often find it difficult to find healthy food choices on food apps,”

“I feel that ordering online from food apps has increased my food intake and appetite,” and “Using online food delivery applications has changed my eating habits (for example: having late night meals, eating alone).” In addition, a total food safety score was calculated similarly, with a higher score indicating more food safety-inclined perceptions. During the data analysis, the score was transformed using a minimum-maximum scaling approach to normalize the data and convert it to a percentage (out of 100). Differences in the perception of healthy food on OFDA and food safety and hygiene according to sociodemographic characteristics and OFDA use were explored using the Mann–Whitney U and Kruskal-Wallis H tests. Pairwise comparisons were conducted to indicate which groups significantly differed from each other. A general linear model analysis was performed to investigate whether certain characteristics (independent variables) can predict the perceptions of healthy food perception and food safety and hygiene scores (score as dependent variable). *p* values at <0.05 were considered statistically significant. Data were analyzed using SPSS software, version 26.0 (SPSS, Chicago, IL, United States).

## 3 Results

### 3.1 Study participants’ characteristics

A total of 532 adolescents participated in the study comprised of 325 females (61.2%) and 207 (38.9%), as shown in Table 1. Most participants were between 17 and 19 years old (40.6%), followed by younger adolescents who were between 14 and 16 years old (35.2%). Most participants lived in Sharjah (32.7%), followed by Abu Dhabi (27.3%). Around a third of the participants had a daily allowance of <25 AED (~7 USD; 37.8%), and 60.5% of the participants reported that their mothers were not employed.

**Table 1:** Sociodemographic characteristics of the study participants (*n* = 532).

Characteristic	n	%
Age (years)		
10–13	129	24.2
14–16	187	35.2
17–19	216	40.6
Sex		
Male	207	38.9
Female	325	61.1
Emirate of residence		
Abu Dhabi	145	27.3
Dubai	113	21.2
Sharjah	174	32.7
Northern Emirates^a^	100	18.8
Daily allowance (AED)		
None	143	26.9
<25 AED	201	37.8
25- < 50 AED	111	20.9
≥50 AED	77	14.5
Mother work		
Yes	210	39.5
No	322	60.5

^a^Northern Emirates including Ajman, Um Al Quwain, Ras Al Khaimah, and Fujairah.

### 3.2 Use of OFDA and healthy food ordering

**Figure 1:**
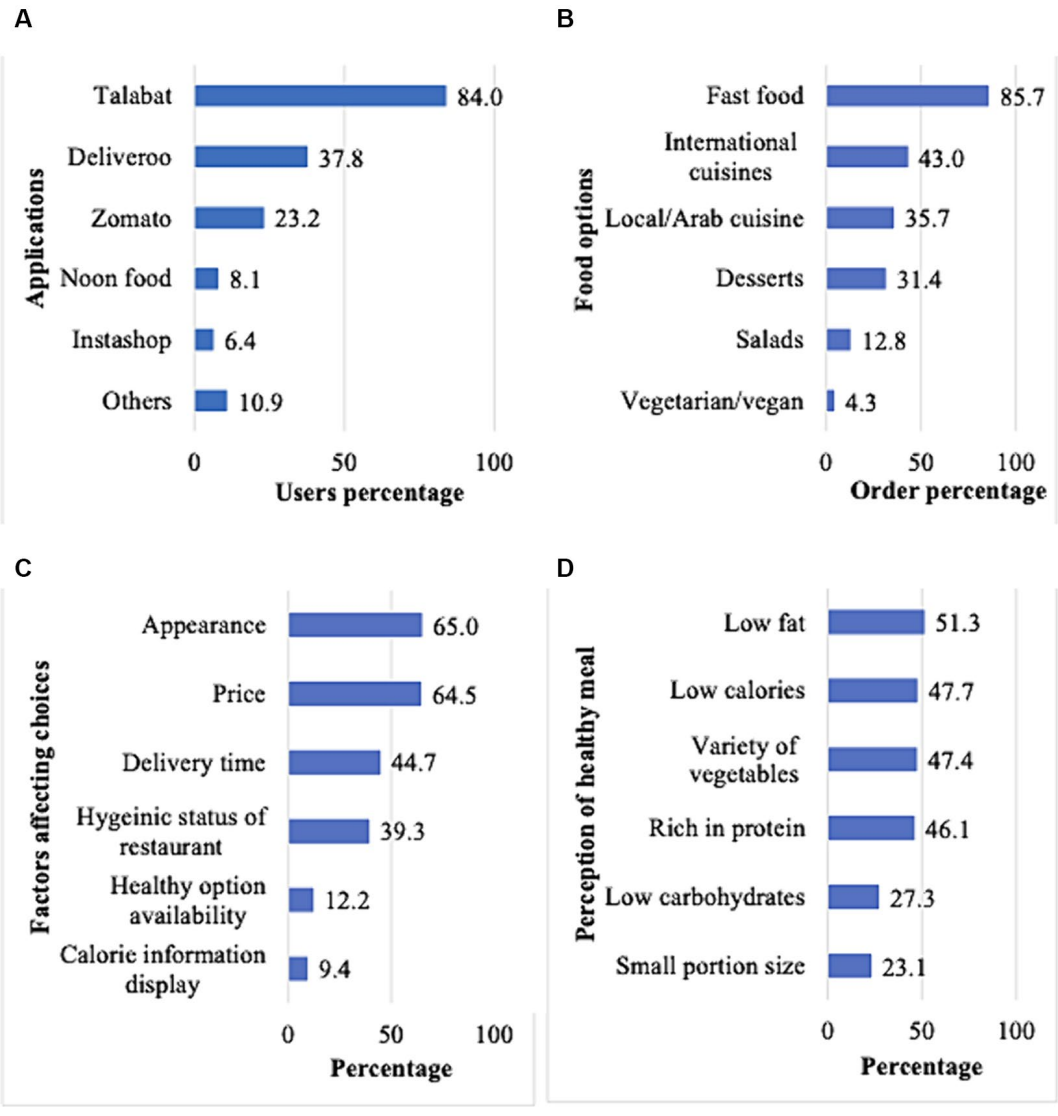
Trends of OFDA among study participants (**n** = 532). **(A)** Most OFDA used (Multiple responses were allowed; others include Uber Eats, Eat Clean me, EatEasy, Careem now, Carriage, and restaurant apps); **(B)** Most ordered cuisine. (Multiple responses were allowed); **(C)** Factors affecting food choice on OFDA among participants; **(D)** Perception of a healthy meal when using OFD applications among participants; Multiple responses were allowed.

Most participants reported using OFDA once per week (35.9%) or 2–3 times per week (29.5%), as shown in Table 2. Approximately three-quarters of the participants reported that there is either always or sometimes food available at home when ordering through OFDA (30.1 and 42.7%, respectively). Figure 1 illustrates the trends of OFDA use among the study participants, where 447 participants reported using Talabat (84.0%), and 456 participants reported mostly ordering fast food (85.7%), followed by 229 participants ordering international cuisines (43.0%) and 190 participants preferring local cuisines (35.7%).

**Table 2:** Use of OFDA and healthy food ordering among the study participants (*n* = 532).

Variable	n	%
Frequency of OFD use		
Daily	21	3.9
4–6 times/week	51	9.6
2–3 times/week	157	29.5
1 time/week	191	35.9
1 time/month	112	21.1
Food at home when ordering		
Yes	160	30.1
Sometimes	227	42.7
No	145	27.3
Look for healthy options on OFDA		
Yes	110	20.7
Sometimes	213	40.0
No	209	39.3
The main concern about ordering healthy food		
Taste	158	29.7
High price	150	28.2
Option availability	92	17.3
Small portion size	69	13.0
Low quality	46	8.6
Appearance	17	3.2

When asked about factors affecting their food choices, most participants were affected by appearance and price (~65%), followed by delivery time (44.7%). Only 20.7% of the participants reported always looking for healthy food options, while 40.0% reported sometimes doing so on OFDA. The most reported concern regarding healthy food ordering was taste (29.7%) and high price (28.2%). When asked about their perception of a healthy meal, almost half of the participants perceived a meal that is low in fat, low in calories, has a variety of vegetables, or is rich in protein (46.1–51.3%).

### 3.3 Perceptions of healthy food on OFDA

**Figure 2:**
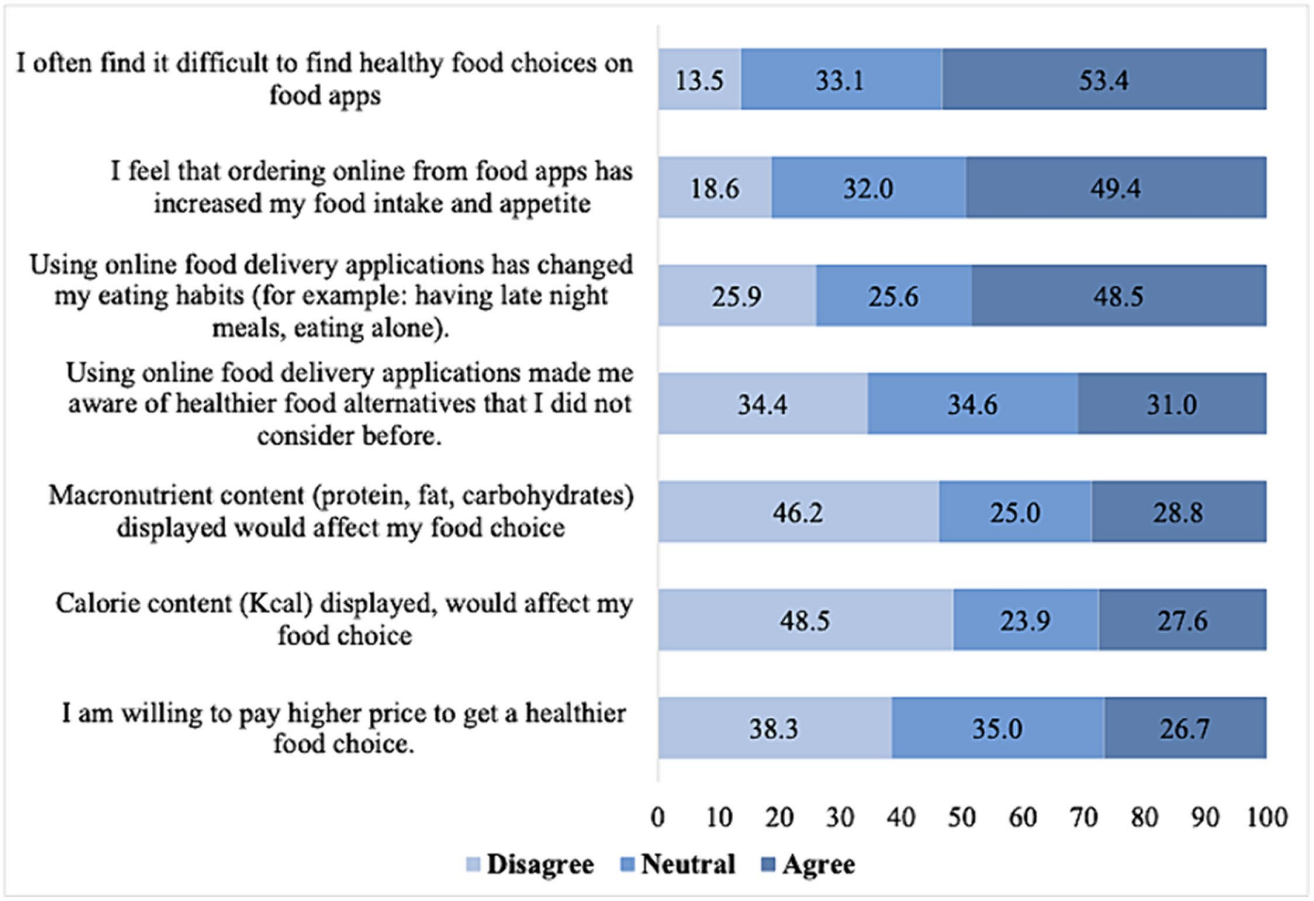
Perceptions of healthy food ordering through the OFD applications (*n* = 532).

Figure 2 shows participants’ agreement on seven statements about placing healthy food orders through OFDA. The participants’ responses were grouped into agree, neutral, and disagree. Two hundred and eighty-four participants (53.4%) agreed on the difficulty of finding healthy food options on OFDA, 263 agreed that the OFDA increased their food intake and appetite (49.4%), while 258 agreed that their eating habits were affected by OFDA (48.5%), specifically in terms of consuming more late-night snacks or eating alone. A lesser proportion of the participants agreed that OFDA made them aware of healthier food alternatives (31.0%), that having calorie and macronutrient content displayed on OFDA might affect their food choices (28.8 and 27.6%), and that they are willing to pay higher prices for healthier food options on OFDA (26.7%).

### 3.4 Perceptions of food safety and delivery hygiene

**Figure 3:**
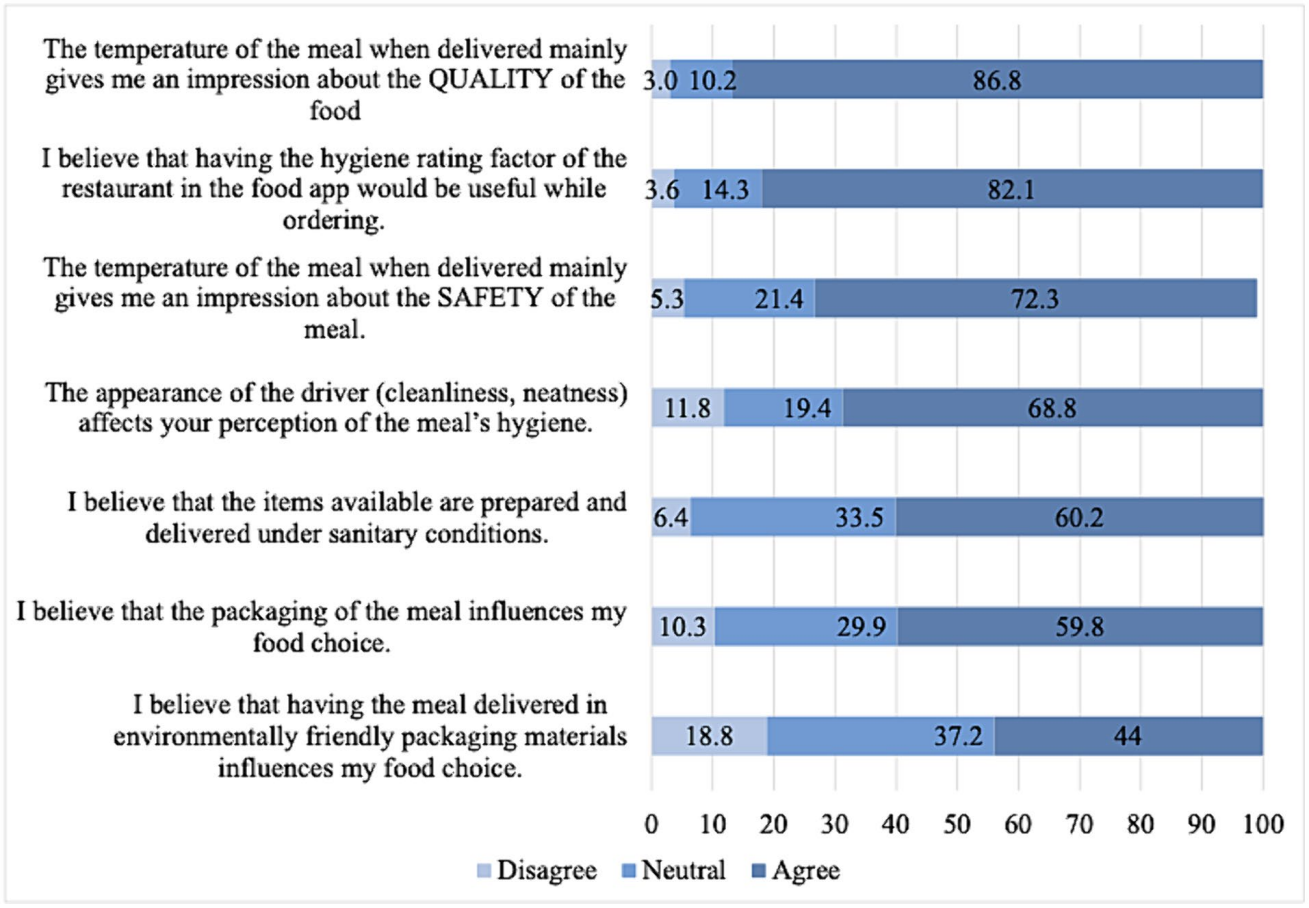
Perceptions of food safety and delivery hygiene through the OFD applications (*n* = 532).

Figure 3 shows participants’ agreement to several statements related to food safety and hygiene of food delivery. Most of the participants agreed that the temperature of the meal upon delivery is a good indicator of both the quality and safety of the food (86.8 and 72.3%, respectively). A similar proportion agreed hygiene ratings would be useful when ordering food (82.1%). Additionally, 68.8% of participants agreed that the driver’s cleanliness and neatness impacted their perception of the meal’s hygiene. Moreover, around 60% agreed that food available through the OFDA was prepared and delivered under sanitary conditions and that packaging influences food choices. Less than half of the participants (44.0%) agreed that using environmentally friendly packaging influences their food choices.

### 3.5 Differences in perception of healthy food and food safety on OFDA

Table 3 presents the differences in the study participants’ perceptions of healthy food, food safety, and hygiene scores. The analysis indicated a significant difference in food safety and hygiene scores (*p* < 0.001), with females having higher scores than males. However, no significant difference in healthy food perception was observed. Moreover, individuals who actively look for healthy food have significantly higher perceptions of healthy food and food safety compared to those who do not actively seek healthy food. Specifically, those who actively seek healthy food have higher healthy food perception scores (*p* < 0.001) and higher food safety scores (*p* = 0.012). The pairwise comparisons further highlight the significance between different groups regarding healthy food perception and food safety scores, specifically emphasizing the impact of actively seeking healthy food on both perceptions.

**Table 3:** The difference in perception of healthy food on OFDA and food safety and hygiene scores (out of 100) according to sociodemographic characteristics and OFDA use (*n* = 532).

		Healthy food perception score			Food safety and hygiene score		
	n (%)	Median	IQR	*p* value	Median	IQR	*p* value
Total		28.6	14.3		39.3	10.7	
Age (years)							
10–13	129 (24.2)	25.0	14.3	0.054	42.9	10.7	0.447
14–16	187 (35.2)	28.6	14.3		39.3	10.7	
17–19	216 (40.6)	28.6	17.9		39.3	14.3	
Sex							
Male	207 (3.9)	28.6	14.3	0.461	39.3	10.7	**0.003**
Female	325 (61.1)	28.6	14.3		42.9	10.7	
Daily allowance (AED)							
None	143 (26.9)	28.6	14.3	0.598	42.9	17.9	0.687
<25 AED	201 (37.8)	28.6	14.3		39.3	10.7	
25- < 50 AED	111 (20.9)	28.6	14.3		39.3	7.1	
≥50 AED	77 (14.5)	32.1	17.9		39.3	14.3	
Mother work							
Yes	210 (39.5)	28.6	10.7	0.126	39.3	10.7	0.804
No	322 (60.5)	28.6	14.3		42.9	14.3	
Frequency							
Frequent	229 (43.0)	28.6	14.3	0.051	39.3	10.7	0.156
Infrequent	303 (57.0)	28.6	17.9		42.9	10.7	
Look for healthy food							
Yes	110 (20.7)	28.6 ^a^	14.3	**<0.001**	46.4 ^a^	17.9	**0.012**
Sometimes	213 (40.0)	32.1 ^b^	14.3		39.3	10.7	
No	209 (39.3)	25.0 ^ab^	14.3		39.3 ^a^	10.7	

*p value was based on the Mann Whitney U test and Kruskal Wallis K test at a 5% level of significance; ^a^pairwise comparison significant difference between Yes and No. ^b^pairwise comparison significant difference between Sometimes and No. Bold values represent significant values based on a *p* < 0.05.

### 3.6 Association between the healthy food perception and food safety and hygiene scores and participants’ characteristics

Table 4 shows the association between several sociodemographic and OFDA use and the healthy food perception score using general linear model analyses. The analysis revealed that the healthy food perception score was significantly lower by 2.6 and 1.9% among younger participants aged 10 to 13 years and frequent users (*B* = −2.6, 95% CI: −4.8–−0.3, *p* = 0.026) and (*B* = −1.9, 95% CI: −3.7–−0.1, *p* = 0.038) respectively. On the other hand, having a working mother and reporting looking for healthy food was associated with a 1.8 and 5.8% significantly higher score (*B* = 1.8, 95% CI:0.1–3.6, *p* = 0.043) and (*B* = 5.8, 95% CI:3.5–8.2, *p* < 0.001) respectively.

**Table 4:** Association between the healthy food perception and food safety and hygiene scores (out of 100%) and participants’ characteristics (*n* = 532).

Parameter	Healthy food perception score			
		95% CI		
	B	Lower	Upper	*p* value
Intercept	28.5	25.6	31.3	**<0.001**
Sex (reference: female)				
Male	−0.5	−2.3	1.3	0.571
Age category (reference: 17-19 years)				
10–13 years	−2.6	−4.8	−0.3	**0.026**
14–16 years	−1.0	−3.0	1.0	0.337
Allowance (reference: ≥50 AED)				
None	−1.3	−4.2	1.5	0.361
<25 AED	−1.2	−4.0	1.5	0.376
25- < 50 AED	−2.6	−5.6	0.4	0.089
Mother work (reference: no)				
Yes	1.8	0.1	3.6	**0.043**
Frequency of use (reference: infrequent)				
Frequent	−1.9	−3.7	−0.1	**0.038**
Look for healthy food (reference: no)				
Yes	5.8	3.5	8.2	**<0.001**
Sometimes	5.7	3.7	7.6	**<0.001**
Parameter	Food safety and hygiene score			
		95% CI		
	B	Lower	Upper	*p* value
Intercept	39.0	36.6	41.5	**<0.001**
Sex (reference: female)				
Male	−2.2	−3.8	−0.7	**0.005**
Age category (reference: 17–19 years)				
10–13 years	0.7	−1.3	2.6	0.507
14–16 years	1.0	−0.7	2.8	0.246
Allowance (reference: ≥50 AED)				
None	0.3	−2.1	2.8	0.785
<25 AED	0.5	−1.9	2.8	0.706
25- < 50 AED	0.7	−1.8	3.3	0.572
Mother work (reference: no)				
Yes	0.4	−1.1	1.9	0.611
Frequency of use (reference: infrequent)				
Frequent	−0.9	−2.5	0.6	0.234
Look for healthy food (reference: no)				
Yes	2.7	0.7	4.7	**0.008**
Sometimes	1.1	−0.5	2.8	0.187

CI, Confidence interval; *p*-values based on a 5% level of significance following general linear model analyses; Dependent variable: Score %. Bold values represent significant values based on a *p* < 0.05.

Regarding the food safety and hygiene score, the analysis revealed that the score was significantly lower by 2.2% among males compared to females (B = −2.2, 95% CI: −3.8–−0.7, *p* = 0.005). On the other hand, looking for healthy food was associated with a 2.7% significantly higher score (*B* = 2.7, 95% CI:0.7–4.7, *p* = 0.008).

## 4 Discussion

The present study provided valuable insights into perceptions of healthy food and food safety and hygiene through OFDA among a sample of adolescent OFDA users in the UAE. Less than half (43.0%) of the participants were frequent users of OFDA (2 times or more/week). This frequency was higher than that of Jordanian adults (35.7%), college students in Malaysia (18.3%), and Brazilian adults (10.0%) (29–31). Among the several OFDA platforms available, Talabat was the most popular choice among our participants, perhaps due to its established popularity and performance in the country and the region (32). Moreover, in the current study, fast food was the dominant choice for orders via OFDA, highlighting adolescents’ tendency toward palatable, convenient, and readily available choices. This is supported by studies in China and Australia, where fast-food outlets comprised 65 and 54% of the available food outlets through OFDA (33, 34).

Available literature points out the adverse impacts of an unhealthy food environment around schools and reveals increased discretionary food purchasing (35) and positive associations with children’s weight status (36). Our findings highlight the need to consider not only food environments around schools but also the threats of the digital food environment, which has facilitated obtaining unhealthy food items with just a few clicks (37).

In the current study, visual appeal and affordability were key drivers for adolescents in making their food choices through OFDA. Similarly, a Polish study on food choices revealed that sensory appeal and price were the prominent drivers of food choice among adolescents (38). In our study, most adolescents reported looking for healthy food options either always or sometimes. This is a favorable find as it indicates a possible growing interest in healthy eating habits among this group. Taste and high prices emerged as key issues regarding healthy food ordering in our sample. This creates a paradox for adolescents, because while healthy food can be available on these apps, the prices are usually outside their budget (39), which can cost up to twice as much as unhealthy food (40). When adolescents buy their food, individual budgetary limitations might influence their food choices, with many food selections being based on meal deals or special food offers (39).

A study published by Fleming et al., including over 600 adolescents from 18 countries globally, revealed that while adolescents were somewhat aware of what a healthy diet is, several factors shape their food options and compromise their intake. Identified factors included taste, cost, and availability of healthy food options which remain barriers limiting their ability to make informed choices (41). Therefore, understanding these food choice drivers may help promote healthy food intake and limit the tendency toward unhealthy food options.

Research shows that adolescents perceive healthy eating to encompass moderation, balance, and variety (42). Perceptions of a healthy meal varied in the present study, with almost half of the participants associating healthiness with low fat, low calories, a variety of vegetables, and rich in protein. On the other hand, perceptions of small portion sizes and low carbohydrates as indicators of healthiness were less reported by our participants. These findings highlight the variable comprehension regarding healthy food among adolescent OFDA users, underscoring the importance of connecting how consumers view healthy eating and investigating their subsequent behaviors to advocate for improved dietary quality (43). Moreover, future research should also look into how adolescents perceive their body weight, which may impact how they eat and their food choices (44, 45).

In this study, perceptions of healthy food on OFDA were investigated, and the findings revealed that almost half of the participants agreed that it is difficult to find healthy options and that their food intake and eating habits have changed after using these apps. The findings point out the well-established lack of availability and the visibility of healthy options through these apps (33, 46).

Further analysis in our study revealed that actively seeking healthy food may positively impact perceptions of healthy food. Therefore, boosting the visibility and the number of healthy food options and implementing calorie declaration beside food items could be effective strategies to enhance the healthiness of people’s food options. Moreover, educating the public on healthy eating can help mitigate the adverse impacts on eating habits associated with using these apps (37, 47). Moreover, in the present study, certain factors, such as older age, less frequent usage, and looking for healthy food options, aligned with a more positive perception of healthy food on OFDA. This suggests that with age, adolescents may gain a better understanding of healthy eating. In addition, given that less frequent users had more positive perceptions of healthy food on these platforms, calling for highlighting the negative impacts of frequent consumption of food away from home and the increased exposure to mostly nutrient-poor and energy-dense foods that may distort their perceptions of healthy food on these apps (48).

In this study, participants mostly agreed on the importance of the temperature of food when delivered as an indicator of the quality and safety of food and the importance of the driver’s cleanliness and demeanor on their perception of the food’s hygiene. These findings highlight the importance of these food delivery hygiene aspects among adolescents, consistent with other studies showing that consumers are concerned about these issues when eating away from home (49, 50). Further analysis in this study revealed that females and those who actively look for healthy options had higher food safety perception scores regarding food safety and hygiene. These findings are supported by research showing that females tend to be more aware and vigilant when choosing safe and hygienic restaurants (51). Moreover, from a restaurant/provider perspective, guaranteeing adherence to food safety regulations and translating this into all food delivery stages can help increase consumers’ trust and recurring ordering (52).

The use of OFDA is becoming more prevalent, with increased use among the youth, given the advancements in technology and current ease of accessibility, raising concerns regarding their potential impact on public health outcomes and their alignment with Sustainable Development Goals (SDGs). While direct evidence linking OFDA to health and nutrition outcomes remains scarce, these platforms play an indirect role by providing convenient access to food (53). Existing research emphasizes the importance of examining the relationship between OFDA and the prevalence of non-communicable diseases (NCDs). Studies have revealed that many menu items available on OFDA fail to meet recommendations for healthy eating, with high levels of saturated fats, trans-fats, free sugars, and salt posing risks for NCDs (46, 54). Concerns are further raised by evidence showing that food options from OFDA tend to contain significantly more calories compared to retail products, potentially contributing to overconsumption and subsequent weight gain (20, 21). Moreover, from an environmental perspective, food ordered from OFDA often comes with high amounts of plastic packaging, such as containers, utensils, and bags, which are disposable and demand considerable energy and resources for production, transportation, and disposal contributing to environmental harm (55–57). However, amidst these concerns, to pave the way toward creating healthier digital food environments, it is suggested that these platforms provide and promote the purchasing of healthy and sustainable food options (58). In addition, researchers suggest collaborative efforts involving reforms in the food industry, coordinated public health communication, and ongoing monitoring of the expanding influence of OFDA could contribute to addressing various interconnected issues such as sustainability, environmental health, and health (59).

To our knowledge, this is the first study to investigate the trends of OFDA usage among adolescent users and their perceptions of healthy food ordering. Despite the cross-sectional design being suitable to fulfill the study objectives, several limitations should be acknowledged. The use of a self-reported questionnaire may lead to social desirability bias or misreporting of data. Moreover, the use of convenience sampling due to difficulties in accessing and recruiting this specific subset of adolescents within a limited timeframe may have limited the generalizability of findings beyond the subset of adolescents who already use OFDA regularly, potentially introducing sampling bias. In addition, the responses were dependent on voluntary participation, which may have led to variations in the representativeness of the different emirates. Moreover, the narrow focus on adolescents who use these apps more frequently may have limited the generalizability and understanding of OFDA use of the broader adolescent population in the country.

## 5 Conclusion

The study highlights the challenges in healthy food accessibility and the use of OFDA among young users. The findings highlight unfavorable food choices among adolescent users, with appearance and price as the main drivers for food choices. It also sheds light on their perceptions of healthy food and their concerns regarding healthy food ordering. These findings highlight critical links between OFDA usage patterns, food choice motivations, and views among adolescents, pushing for tailored interventions to promote healthier food choices and improve food safety perceptions. To date, the actual impact of OFDA on health remains unclear. However, research is focusing on the digital food environment and how it can be used to improve people’s dietary habits and overall well-being. Moreover, a holistic and sustainable approach is necessary, considering the SDGs, current environmental and economic constraints, and the complex influences on behavior. Collaboration among OFDA platforms, food vendors, and regulators can promote the availability of healthy, sustainable options, incentivize eco-friendly practices, and implement pricing strategies to enhance affordability. Education campaigns and regulatory measures can raise awareness and create a supportive environment for healthy choices. Ultimately, ongoing research and evaluation efforts are essential to understand the long-term impacts of these interventions and ensure their effectiveness in promoting sustainable dietary behaviors among adolescents.

## Data availability statement

The datasets presented in this study can be found in online repositories. The names of the repository/repositories and accession number(s) can be found at: https://figshare.com/s/9471b272877522041f79.

## Ethics statement

The studies involving humans were approved by University of Sharjah Research Ethics Committee (REC-22-02-16-09-S). The studies were conducted in accordance with the local legislation and institutional requirements. Written informed consent for participation in this study was provided by the participants’ legal guardians/next of kin.

## Author contributions

SS: Conceptualization, Formal analysis, Investigation, Writing – original draft, Writing – review & editing. TO: Conceptualization, Methodology, Writing – review & editing. AA-J: Writing – review & editing. HH: Writing – review & editing. MH: Writing – review & editing. MM: Formal analysis, Methodology, Writing – original draft, Writing – review & editing. SQ: Investigation, Writing – review & editing. HS: Writing – review & editing. RD: Investigation, Writing – review & editing. RR: Investigation, Writing – review & editing. EM: Formal analysis, Writing – review & editing. LS: Writing – review & editing. DP: Writing – review & editing. AZ: Writing – review & editing. AD: Writing – review & editing. HK: Investigation, Writing – original draft. LCI: Conceptualization, Methodology, Supervision, Writing – review & editing.
